# Chitosan Hydrogel Enhances the Therapeutic Efficacy of Bone Marrow–Derived Mesenchymal Stem Cells for Myocardial Infarction by Alleviating Vascular Endothelial Cell Pyroptosis

**DOI:** 10.1097/FJC.0000000000000760

**Published:** 2019-11-11

**Authors:** Yang Liu, Panyang Li, Chenhui Qiao, Tiejun Wu, Xiaoke Sun, Meng Wen, Weihua Zhang

**Affiliations:** * Department of Cardiovascular Surgery, The First Affiliated Hospital of Zhengzhou University, Zhengzhou, China; and; † Department of Anatomy, Faculty of Basic Medicine, Henan Medical College, Zhengzhou, China.

**Keywords:** myocardial infarction, mesenchymal stem cells, hydrogel, bioluminescence imaging

## Abstract

Myocardial infarction (MI) is one of the higher mortality rates, and current treatment can only delay the progression of the disease. Experiments have shown that cell therapy could improve cardiac function and mesenchymal stem cells (MSCs)-based therapies provide a great promising approach in the treatment of MI. However, low cell survival and engraftment restricts the successful application of MSCs for treating MI. Here, we explored whether co-transplantation of a chitosan (CS) thermosensitive hydrogel with bone marrow-derived MSCs (BMSCs) could optimize and maximize the therapeutic of BMSCs in a mouse model of MI. The fate of transplanted BMSCs was monitored by bioluminescence imaging, and the recovery of cardiac function was detected by echocardiogram. Our results proved that CS hydrogel enhanced the BMSCs' survival and the recovery of cardiac function by protecting the vascular endothelial cells. Further studies revealed that the increased number of vascular endothelial cells was due to the fact that transplanted BMSCs inhibited the inflammatory response and alleviated the pyroptosis of vascular endothelial cells. In conclusions, CS hydrogel improved the engraftment of transplanted BMSCs, ameliorated inflammatory responses, and further promoted functional recovery of heart by alleviating vascular endothelial cell pyroptosis.

## Abstract 2

Supplemental Digital Content is Available in the Text.

## INTRODUCTION

The incidence of ischemic heart disease has increased in recent years; however, myocardial infarction (MI) is the most common cause of mortality and disability.^1^ MI usually leads to the ischemic condition including a decrease in the supply of oxygen and nutrients, causing necrosis of cardiomyocytes and endothelial cells.^2^ After MI, the inflammatory response and subsequent heart tissue repair are inevitable to the body.^3^ Proper inflammatory responses have certain benefits to the body, and secreted cytokines may promote tissue angiogenesis. However, excessive inflammatory reactions can cause the inappropriate repair for damaged tissue and formation of scar tissue.^4^ Therefore, it is necessary to maintain the balance between the inflammatory responses and tissue repair. Otherwise, the inflammatory process will be enlarged, further resulting in damage and abnormal repair, and eventually will lead to cardiac remodeling and decreased cardiac function.^5^

Previous research has shown that mesenchymal stem cells (MSCs) not only had the self-renewal and multidirectional differentiation potential but also had the strong immunomodulatory effects, which could inhibit inflammatory response, prolong graft survival time, and reduce graft-versus-host disease.^6^ In addition, transplanted MSCs exerted the anti-inflammatory effects, thereby improving left ventricular function post-MI.^7^ MSCs do not express major histocompatibility complex class II molecules, so they are not immunogenic. However, MSCs could reduce the release of harmful inflammatory factors by immunosuppression and could secrete prostaglandin E2 (PGE2) under the induction of inflammatory environment.^8^ Prostaglandin E2 further promotes the secretion of anti-inflammatory factor interleukin-12 (IL-12) and interleukin-10 (IL-10). IL-10 can relieve the harmful inflammatory responses and accelerate wound healing.^9^ Moreover, when MSCs were injected into the wound surface, the reduced expression of proinflammatory factors interleukin-1β (IL-1β) and interleukin-6 (IL-6) in the wound tissue could be observed, whereas the expression of the anti-inflammatory factor IL-10 was increased, demonstrating MSCs could promote wound healing by reducing the local inflammatory responses of the wound.^10^

Immunocyte-mediated tissue inflammation is increasingly recognized as a key factor in organ damage.^11^ The cell inflammatory death-pyroptosis, a new form of programmed cell death, is an important part of innate immunity and is involved in the development of many cardiovascular diseases.^12^ Pyroptosis is a special programmed cell death triggered by activated caspase-1.^13^ The inflammatory responses of body activate caspase-11 or NOD-like receptor family pyrin domain containing 3 (NLRP3) inflammasome, and the proteolytic activation of NLRP3 promotes the precursor cleavage of caspase-1, which further cleaves the proinflammatory cytokine interleukin-1β (IL-1β) and interleukin-18 (IL-18) to make it a mature form.^13,14^ Caspase-1 or caspase-11–mediated cleavage of the protein gasdermin D (GSDMD) produces an aminoterminal fragment that induces the pyroptosis. And GSDMD controls the release of mature IL-1β and IL-18, accompanied by host cell death.^13,14^

Recent studies have shown that pyroptosis-related proteins caspase-1 and IL-1β were significantly upregulated in ischemic animal models.^15^ Using the immunosuppressive effects of MSCs to arrest cell death in MI has become a promising treatment.^16^ However, the low survival rate after stem cell transplantation greatly limits its application.^17^ Engineered biological matrices can mimic the microenvironment of cells in living organisms to provide a post-transplant platform for stem cells.^18–20^ In our study, we hypothesized that chitosan (CS) hydrogel could increase the survival of bone marrow–derived MSCs (BMSCs) and have a better therapeutic effect on tissue repair. Hence, we explored the therapeutic effects of BMSCs co-transplanted with CS hydrogel in a murine MI model. We monitored the fate of BMSCs by using molecular imaging approaches. Furthermore, we investigated the effect of BMSC-derived anti-inflammatory responses and proangiogenesis by suppressing the pyroptosis of vascular endothelial cells post-MI.

## METHODS

### Cell Culture

BMSCs were isolated from transgenic mice expressing firefly luciferase (Fluc) and green fluorescent protein (GFP) constitutively as reported methods previously.^21^ BMSCs were cultured in Dulbecco's Modified Eagle Medium/F12 (Gibco, Grand Island, NY) supplemented with 10% fetal bovine serum (FBS; HyClone, Smithfield, Australia), 1% penicillin-streptomycin (Gibco), 1% mM nonessential amino acids (Gibco), and 1% NEAA (Gibco). Human umbilical vein endothelial cells (HUVECs) purchased from the American Type Culture Collection (Manassas, VA) were maintained in endothelial growth medium-2 (EGM-2; Lonza, Walkersville, MD). For the collection of the BMSCs' conditioned medium (CM), CM was collected from P6 BMSCs. Briefly, cells were cultured on the plates or coated CS hydrogel plates at a proper density and allowed to grow for 3 days in a 5% humidified CO_2_ atmosphere at 37°C. The culture medium was then renewed and collected 24 and 96 hours thereafter (cell culture was not renewed or added during this time period). Collected CM were frozen and thawed only in the day of experiments.

### CCK-8 Assay

To investigate the optimal concentration of CS hydrogel for BMSCs' viability, the 1 × 10^4^ BMSCs were seeded into 96-well plates coated with different concentration of CS hydrogel (0.5, 1, 1.5, and 2 mg/mL) for 24 hours. Then, the reagent of Cell Counting Kit-8 (CCK-8; Dojindo Molecular Technologies, Rockville, MD) was used to treat with the cells and incubated for another 2 hours. The absorbance value of each sample at 450 nm was recorded with the microplate reader (Thermo Labsystems, Vantaa, Finland). To investigate the effect of CS hydrogel on BMSCs' proliferation, 96-well plates were coated with 1 mg/mL CS hydrogel and 1 × 10^4^ BMSCs were added per well. The CCK-8 assay was performed at the specific point in time (24, 48, 72, and 96 hours).

### Hydrogel Preparation

In this experiment, CS hydrogel was prepared in a similar manner, as previously described.^22^ The 100-mg ultra-pure CS with a 90% degree of deacetylation (M = 200,000; Haidebei Bioengineering Company, Jinan, China) was dissolved in 10-mL HCL (0.1 M) stirred overnight. Then, 1 L of dialyzed water was used to dialyze the CS hydrogel with membrane (molecular cutoff of 8 kDa–10 kDa) for 5 days, ensuring the removal of residual acetic acid. Finally, it was lyophilized to obtain CS hydrochloride. The CS hydrochloride was dissolved in distilled water and added the β-GP solution (Sigma Aldrich, St. Louis, MO) to the solution drop by drop while stirring on ice. The final concentration of β-GP was 0.7% wt/vol. CS hydrogel of different concentrations (0.5, 1, 1.5, and 2 mg/mL) was prepared in this way.

### Bioluminescence Imaging

Fluc activity within different cell numbers and the fate of transplanted cells were confirmed using a Xenogen IVIS Luminar Imaging System (Xenogen Corporation, Hopkinto, MA). In brief, after intraperitoneal injection of the reporter probe D-luciferin (120 mg luciferin/kg body wt, Calipers, Hopkinton, MA), animals were imaged for 1–10 minutes using the IVIS Luminar Imaging System. Following analyzed the signal intensity by utilizing the software of living image. Average radiance of peak bioluminescence imaging (BLI) signal was quantified by from a fixed-area region of interest over the thorax.

### Real-Time PCR

To test the mRNA expression levels of the genes, a TRIzol reagent (Invitrogen, Carlsbad, CA) was used to extract the total RNA, and first-strand cDNA was synthesized by reverse transcriptase (TransGen Biotech, China) with oligo dT primers. The expression of mRNA lever was determined from the threshold cycle normalized to the expression of Gapdh using TransStart Green qPCR SuperMix Kit (TransGen Biotech). Real-time PCR analysis was performed on the Opticon System (Bio-Rad, Hercules, CA). The 2^−ΔΔCt^ method was used to determine the relative mRNA folding changes. Primers are listed in **Supplemental Digital Content 1** (see **supplementary Table 1**, http://links.lww.com/JCVP/A430).

### Echocardiographic Examination

Each group of mice underwent echocardiography on the 30th day post-MI. After anesthesia in mice, the hair in the anterior region of the mouse was removed. The left ventricular short-axis section was examined using two-dimensional ultrasound, and the movement of the left ventricle was detected by M-mode ultrasound. Left ventricular end-diastolic diameter (LVIDd), left ventricular end-systolic diameter (LvIDs), left ventricular ejection fraction (EF), and fractional shortening (FS) were measured. All data were processed using the Vevo 770 V3.0.0 software (VisualSonics Inc, Toronto, Canada) under the same parameters, and each measurement value was the average of 3 consecutive cardiac cycles.

### Immunofluorescence Assay

After the mice were euthanized, the heart sample was harvested and fixed. Immunofluorescence staining was performed to determine the expression of CD31 (ab9498; Abcam, Cambridge, MA) and Caspase-1 (ab1872; Abcam) at the injured sites. Alexa Fluor 488 goat anti-rabbit IgG (CA11008s) and 594 goat anti-mouse IgG (CA11005s) (Invitrogen) were applied. The cell nuclei were counterstained with DAPI (Southern Biotech, Birmingham, AL). Images of 6 randomly selected fields from the infarct and border area were captured using a fluorescence microscope (Zeiss, Oberkochen, Germany).

### Western Blot Analysis

For protein analysis, all the cell extracts (50–100 mg) were lysed with (radio-immunoprecipitation assay) RIPA lysis buffer (Cowei, Beijing, China) containing proteinase inhibitors on ice for at least 0.5 hours. The BCA Protein Assay Kit (Promega, Madison, WI) was used to quantify the concentration of proteins, as previously.^23^ Three independent replicates of 30-μg sample were applied to detect the protein expression level with a primary antibody. The primary antibodies used include GSDMD (1:1000, ab210070; Abcam), caspase-1 (1:1000, ab207802; Abcam), and GAPDH (1:10,000, ab181602; Abcam).

### Animals

Transgenic FVB-Fluc/GFP mice were purchased from the laboratory animal center of the Academy of Military Medical Science (Beijing, China), and this transgenic mouse was made by pronuclear injection of the reporter gene construct in the Beijing Biocytogen and kept in a specific pathogen free environment.^24^ To establish a model of acute MI, the left anterior descending coronary artery of mice was permanently ligated, as previously described.^19^ Mice received cells, phosphate buffer saline (PBS), or CS after the ligation. 2 × 10^5^ BMSCs suspended in 20-μL CS hydrogel (1 mg/mL) or PBS were injected into 2 positions adjacent to the infarcted areas with a 30-gauge needle. The same procedure was performed without ligation for sham-operated control. All the operation conformed to the Guide for the Care and Use of Laboratory Animals published by the US National Institutes of Health.^25^

### Statistical Analysis

All experiments were repeated at least 3 times, and data are expressed as the mean–standard error of measurement (SEM). An independent *t* test was used for two-group comparisons, and one-way analysis of variance, followed by the Student–Newman–Keuls multiple-group comparison. For statistical analysis, Graphpad Prism software version 5.0 was performed to analyze the quantitative data. *P* < 0.05 was defined as significance difference.

## RESULTS

### CS Hydrogel Promotes the Proliferation of BMSCs

**FIGURE 1.**
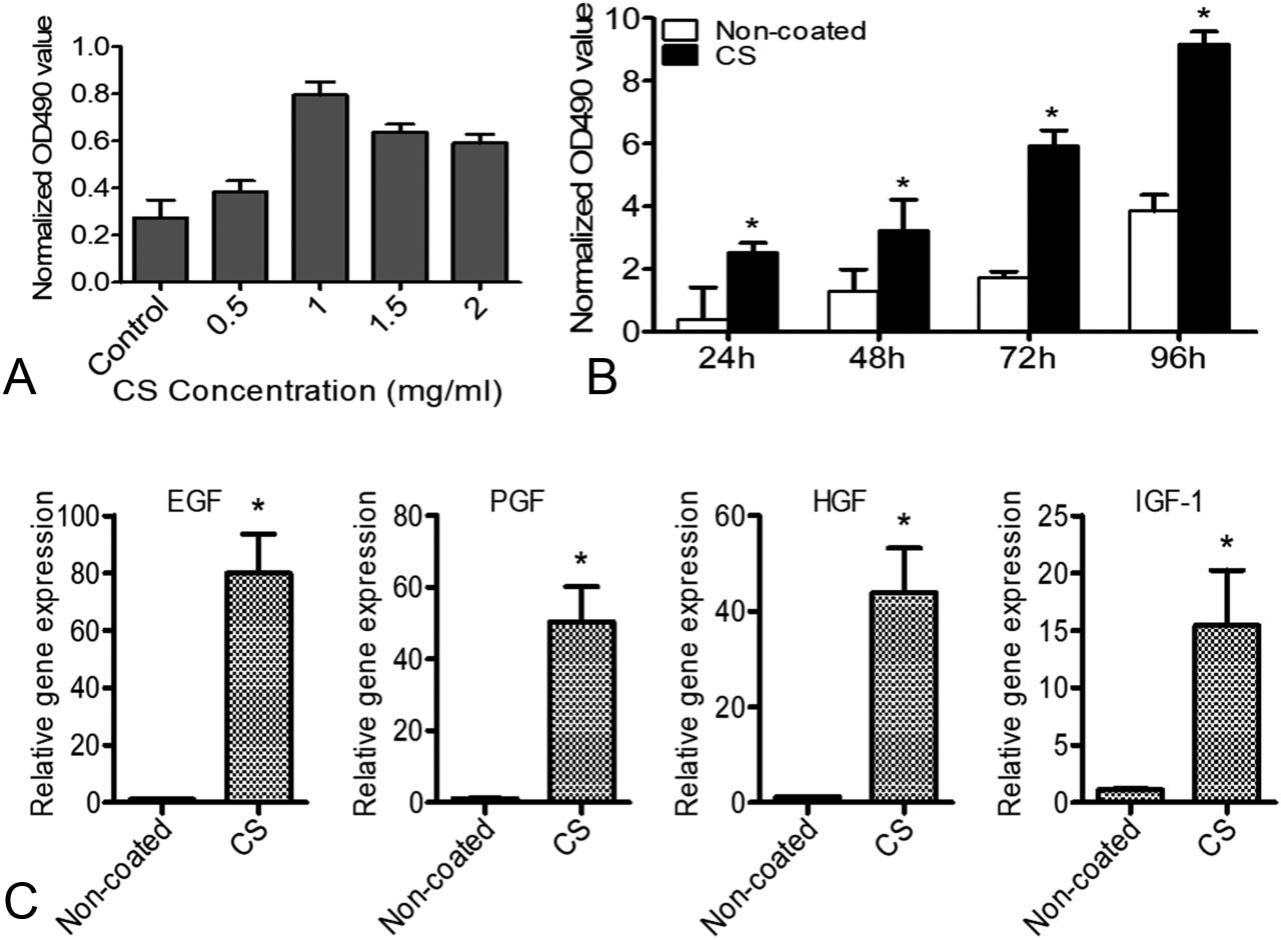
Characterization of CS hydrogel. A, The viability of BMSCs cultured in different concentrations of CS hydrogel was detected by Cell Counting Kit-8 assay in vitro. B, Cell proliferation experiments showed that CS hydrogel promoted the proliferation of BMSCs. Data are expressed as mean ± SEM. *P < 0.05 versus noncoated. C, Real-time RT-PCR analysis of proliferation-related gene expression of BMSCs cultured on CS hydrogel–coated plates for 48 hours. Data are expressed as mean ± SEM. *P < 0.05 versus noncoated. All experiments were performed in triplicate. EGF, epithelial growth factor; HGF, hepatocyte growth factor; IGF, insulin-like growth factor-1; PGF, placental growth factor.

To monitor transplanted cells in real time, BMSCs expressing Fluc and GFP were isolated from transgenic mice. In this experiment, the CS hydrogel was thermosensitive based on the previously reported studies.^26^ The Cell Counting Kit 8 (CCK-8) assay was conducted to detect the bioactivities of CS hydrogel. The proliferation experiment results showed that 1 mg/mL was the optimal concentration for CS hydrogel to promote BMSCs proliferation (Fig. 1A). When BMSCs were cultured on the plate-coated CS hydrogel, cell experiments revealed that BMSCs expanded more rapidly compared with culture on noncoated plates (Fig. 1B). Furthermore, the expression of proliferation-related genes including epithelial growth factor, placental growth factor, hepatocyte growth factor, and insulin-like growth factor-1 was detected by quantitative real-time PCR, showing the all upregulated expression of BMSCs cultured on plate-coated CS hydrogel compared with the noncoated plates (Fig. 1C).

### CS Hydrogel Improved the Engraftment of BMSCs

**FIGURE 2.**
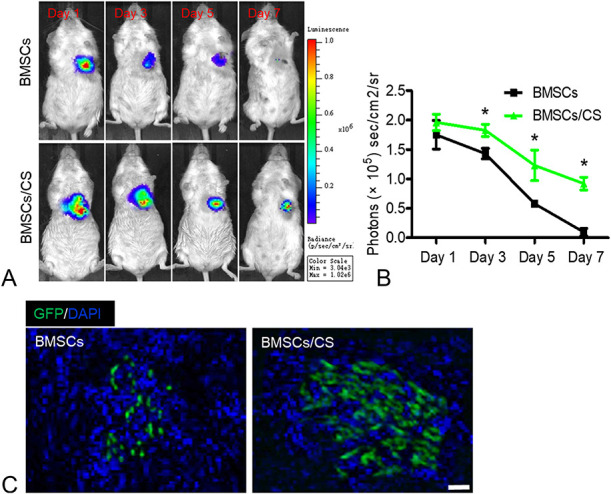
CS hydrogel increases the retention of BMSCs after transplantation. A, The fate of BMSCs after transplantation was tracked by BLI. Representative images of mice transplanted with BMSCs and BMSCs/CS hydrogel. B, Quantitative analysis of BLI signals revealed that cell survival was improved by CS hydrogel. Data are expressed as mean ± SEM. n = 8. *P < 0.05 versus BMSCs. C, Immunofluorescence assay showed the BMSCs (GFP, green) were transplanted into heart tissue on day 5. The cell nucleus (blue) was stained with 4′,6-diamidino-2-phenylindole. All experiments were performed in triplicate. Scale bars, 100 μm.

To assess the engraftment of BMSCs, an MI model was induced by ligation of left anterior descending coronary artery.^27^ After treatment with BMSCs and BMSCs co-transplanted with CS hydrogel, BLI was used to track the survival of BMSCs every 3 days after transplantation. The data showed that the signals of the heart region were strong after cell transplantation on day 1 in each group, indicating successful BMSC transplantation. However, the BMSCs group experienced substantial donor cell death in the following days. A series of BLI signals from the same animal revealed that the application of CS hydrogels improved BMSC transplantation. It is implied that CS hydrogels could dramatically expand the cell survival, thereby possibly impacting the therapeutic potential of BMSCs in MI (Figs. 2A, B). Moreover, we performed immunohistology experiments on heart tissues harvested 5 days after the cell transplantation (Fig. 2C). The results showed that the number of positive cells in BMSCs co-transplanted with CS hydrogel was much higher than the only BMSCs transplantation group, further proving that CS hydrogel could increase the BMSCs engraftment in a MI model.

### Enhanced Effects of Repairing Heart Function

**FIGURE 3.**
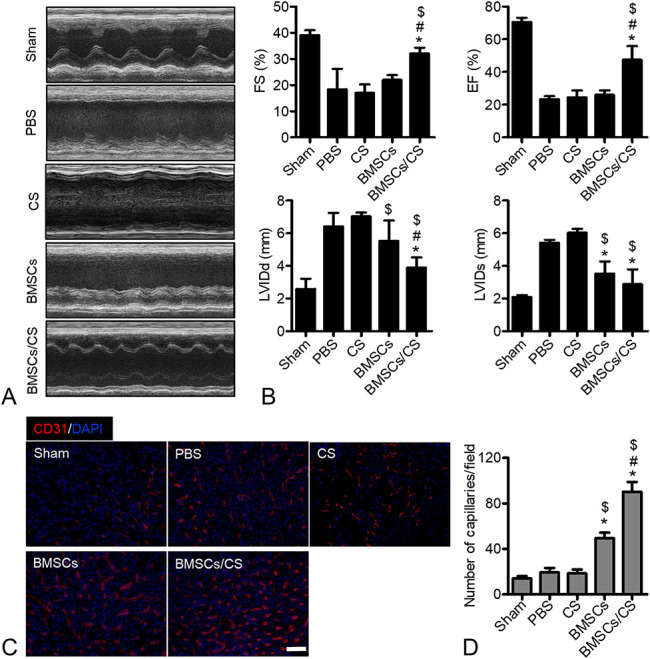
Cotransplantation of BMSCs with CS hydrogel promotes the recovery of heart function. A, M-mode echocardiographic photograhs from the group of sham, PBS, CS, BMSCs, and BMSCs/CS at day 30. B, The levels of EF%, FS%, LVIDd, and LVIDs were evaluated by echocardiography (n = 12). These results suggest that cotransplantation of BMSCs with CS hydrogel could significantly improve cardiac function. Data are expressed as mean ± SEM. n = 8. *P < 0.05 versus PBS, #*P* < 0.05 versus BMSCs, $*P* < 0.05 versus CS. Experiments were performed in triplicate. C, Representative immunofluorescence photographs of CD31 (red) staining to reveal the capillaries in the border region at day 30. D, Quantitative analysis reveal the capillary density. Compared with the PBS and CS group, transplantation of BMSCs with CS hydrogel significantly increased vessel density. Data are expressed as mean ± SEM. n = 8. *P < 0.05 versus PBS, #*P* < 0.05 versus BMSCs, #*P* < 0.05 versus BMSCs, $*P* < 0.05 versus CS. Scale bars, 100 μm. Experiments were performed in triplicate.

After 30 days of cell transplantation, we detected the LV function in each treatment group by echocardiography to evaluate the therapeutic effects of BMSCs in mouse MI. In this experiment, we focused on 4 parameters containing the LVIDd, LVIDs, FS, and EF. Compared with the sham group, the LV of mice in the other treatment groups showed significant enlargement, and the anterior and posterior walls of the LV were significantly thinned. Among them, the PBS and CS group had the worst heart function, and the ventricular contraction ability was poor (Fig. 3A). Through further data analysis of cardiac function it could be found that BMSCs cotransplanted with CS hydrogels could significantly decrease the LVIDd and LVIDs of hearts after infarction compared with PBS, CS, and BMSCs groups (Fig. 3B). At the same time, comparing to PBS, CS, and BMSCs groups, BMSCs cotransplanted with CS hydrogels could obviously maintain the LV contractile function including the increased FS and EF (Fig. 3B). Moreover, to assess the neovascularization in the ischemic myocardium at day 30, capillaries in the border zone were examined by CD31 staining. The results showed that BMSCs cotransplanted with CS hydrogel significantly increased the capillary density in the border region compared with other groups (Figs. 3C, D). Taken together, these findings highlighted the potential of BMSCs cotransplanted with CS hydrogel could effectively alleviate the LV remodeling and significantly restore LV contractile function by enhancing neovascularization after infarction.

### Increased Inflammatory Inhibition Subsequently Prevented the Pyroptosis of Vascular Endothelial Cells

**FIGURE 4.**
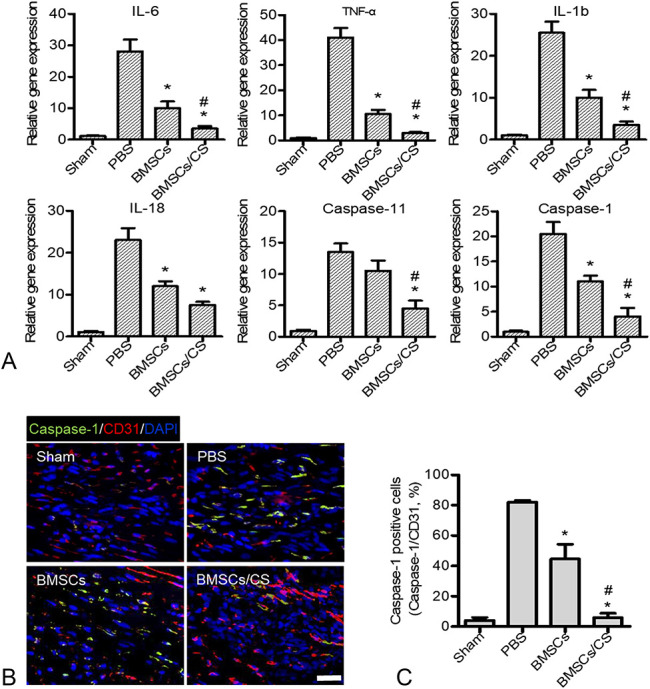
Cotransplantation of BMSCs with CS hydrogel inhibited the inflammatory response and pyroptosis of vascular endothelial cells at the injured sites. A, Real-time PCR analysis of inflammatory-related gene (IL-6, TNF-α, IL-1β, IL-18, caspase-11, and caspase-1) expression at day 5 after MI. Data are expressed as mean ± SEM. *P < 0.05 versus PBS; #*P* < 0.05 versus BMSCs. B, Representative images show caspase-1 immunostaining (green) detected in vascular endothelial cells (red) at day 5. C, Quantitative analysis revealed that cotransplantation of BMSCs with CS could ameliorate the pyroptosis of vascular endothelial cells. Data are expressed as mean ± SEM. n = 8. *P < 0.05 versus PBS; #*P* < 0.05 versus BMSCs. Scale bars, 100 μm. Experiments were performed in triplicate.

Tissue damage, repair, and remodeling after MI are often accompanied by a great number of inflammatory responses. Long-term inflammatory responses will lead to loss of cardiomyocytes, inhibition of systolic function, enlargement of the lumen, loss of ventricular wall integrity, and heart rupture. Increasingly, evidences show that MSCs regulate the immune system by suppressing inflammatory responses by several soluble factors. Next, we explored the transplanted BMSCs-mediated regulation of inflammatory responses detecting the level of tissue inflammatory factors after MI. The results showed that BMSCs cotransplanted with CS hydrogel could significantly reduce the secretion of the inflammatory mediators including IL-6, TNF-α, IL-1β, IL-18, caspase-11, and caspase-1 on day 5 after cell transplantation compared with PBS and BMSCs. In addition, the BMSCs group significantly decreased the secretion of inflammatory factors of IL-18, IL-6, IL-1β, and caspase-1 compared with the PBS group (Fig. 4A). The injury of vascular endothelial cells caused by oxidative stress in inflammatory response is an important factor for affecting tissue repair after MI. Pyroptosis is a programmed inflammatory cell necrosis that is dependent on caspase-1 and is accompanied by the release of numerous proinflammatory factors. To investigate the mechanism by which BMSCs inhibited inflammatory responses and promoted angiogenesis, we performed double immunofluorescence staining of CD31 and caspase-1 on days 7 after treatment (Figs. 4B, C). The results showed that BMSCs cotransplanted with CS hydrogel appeared the less expression of caspase-1 in vascular endothelial cells compared with the PBS and BMSCs group; however, there was a large amount of caspase-1 expression in CD31 positive cells of the PBS group. These results indicated that transplanted BMSCs might reduce the pyroptosis of vascular endothelial cells by inhibiting the inflammatory response after MI, thereby promoting the recovery of cardiac function, which was significantly strengthen in BMSCs co-transplanted with CS hydrogel groups.

### BMSCs Alleviated the Pyroptosis of HUVECs by Paracrine Action

**FIGURE 5.**
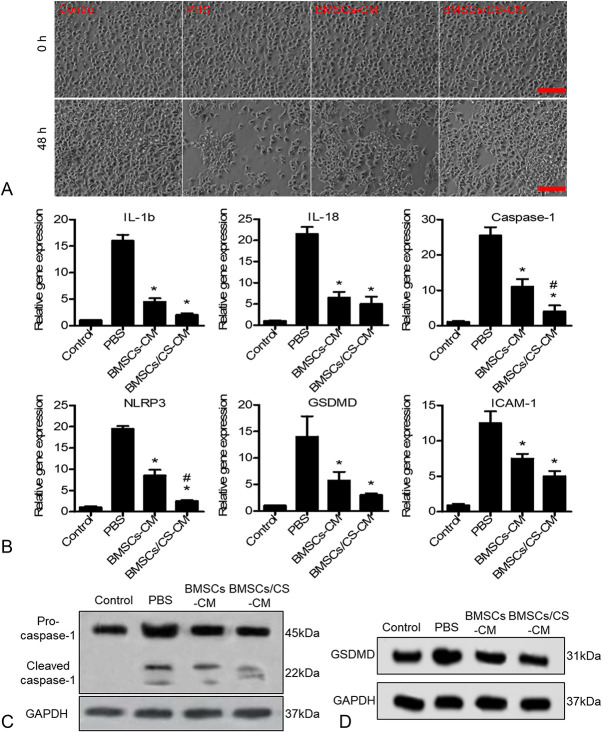
CM of BMSCs can inhibit the pyroptosis of HUVECs induced by LPS and ATP. BMSCs were cultured on CS hydrogel–coated or noncoated plates for 48 hours, and the CM was collected. HUVECs were treated with LPS 6 hours, and then the CM was replaced. A, After 48 hours, we observed the survival of HUVECs under the microscopy. B, After 48 hours, real-time PCR analysis of the expressions of pyroptosis-related gene (IL-1β, LI-18, caspase-1, NLRP3, GSDMD, and ICAM-1). Data are expressed as mean ± SEM. *P < 0.05 versus PBS; #*P* < 0.05 versus BMSCs-CM. C, D, Western blot analysis of the expression of caspase-1 and GSDMD. All experiments were performed in triplicate. BMSCs-CM, BMSCs CM; BMSCs/CS-CM, CM of co-culture BMSCs with CS hydrogel.

To further investigate the mechanism by which transplanted BMSCs prevented the pyroptosis of vascular endothelial cells and promoted cardiac function recovery, the HUVECs were stimulated with lipopolysaccharid (LPS) (10 ng/mL) for 18 hours and then incubated with adenosine triphosphate (ATP) (3 mM) for 1 hour. After 48 hours of treatment with HUVECs in a BMSCs conditioned medium (BMSCs-CM) or CM of co-culture BMSCs with CS hydrogel (BMSCs/CS-CM), we observed the survival of HUVECs and detected the expression of genes associated with pyroptosis in HUVECs by q-PCR. We found that compared with the PBS group, the BMSCs/CS–CM group remarkably improved the survival of HUVECs (Fig. 5A). RT-PCR results showed that pyroptopsis gene expression in the BMSCs–CM group was significantly reduced compared with the PBS group, and this effect was further enhanced in the BMSCs/CS–CM group (Fig. 5B). Because the activation of many caspase enzymes is accompanied by functional maturation of autolytic partial fragmentation, a procaspase-1 and cleaved caspase-1 will eventually form.^28^ In addition, Western blotting results showed that caspase-1 has indeed been activated and BMSCs/CS–CM did inhibit the expression of the pyroptosis key proteins caspase-1 and GSDMD (Figs 5C, D; see **Supplemental Figures 1A and B**, **Supplemental Digital Content 1**, http://links.lww.com/JCVP/A430). These findings revealed that BMSCs could inhibit site inflammatory responses and protect vascular endothelial cell from the pyroptosis by paracrine effects, and CS hydrogel enhanced the effects of BMSCs and promoted cardiac repair.

## DISCUSSION

In this study, we generated the CS hydrogel as a carrier system for BMSCs to treat MI. We found that CS hydrogel could improve the engraftment of transplanted BMSCs and enhance its therapeutic effects of ameliorating heart-remodeling after MI. We further explored the therapeutic mechanisms of transplanted BMSCs with CS hydrogel and showed that transplanted BMSCs could inhibit the inflammatory responses at the injury sites and improve the angiogenesis in MI heart. Transplanted BMSCs with CS hydrogel could regulate the inflammatory microenvironment by enhanced paracrine effects and further alleviate the pyroptosis of vascular endothelial cells.

MI remains the leading cause of cardiovascular-associated mortality and morbidity.^29^ In recent years, many studies have demonstrated that MSC transplantation strategies could effectively improve cardiac function and alleviate symptoms after acute MI.^30^ However, transplanted MSCs in the heart infarct area due to contraction of the heart, or in the ischemic environment and other reasons lead to easy loss and death, resulting in lower survival and resident rate.^30^ These problems seriously weaken the therapeutic effect of the MSCs transplantation strategy. To solve this problem, many kinds of stem cell transplantation carrier bioactive materials have emerged.^31^ Extensive experiments have shown that bioactive materials could not only promote the survival of stem cells in vivo but also improve the function of stem cells.^31–33^ The preparation of a material carrier, such as by simulation or the use of an extracellular matrix, provides a good microenvironment for the survival of MSCs in the MI region.^34,35^ In our experiment, we used CS hydrogel-loaded BMSCs to transplant into the MI region and detected the fate of transplanted BMSCs in real time by molecular imaging. It was found that the application of CS hydrogel promoted the survival of transplanted BMSCs and thus enhanced the efficacy of BMSCs in the treatment of MI. Previous studies indicated that CS hydrogel increases MSC retention, promotes MSC differentiation into myocytes, and increases the effects of MSCs on neovasculature formation. However, other mechanisms require future investigation for clarification.

MI is associated with a sterile inflammatory response.^36^ This inflammatory process is a prerequisite for tissue healing but may also cause excessive damage and maladaptive ventricular remodeling leading to impaired myocardial function and heart failure.^37,38^ Accumulating evidence indicates that the myocardial response to tissue injury is regulated by the innate immune system, involving several families of pattern recognition receptors.^37,38^ It is often accompanied by excessive activation of the immune system, causing a large number of proinflammatory cytokines, such as tumor necrosis factor-α (TNF-α), interleukin-1β (IL-1β), interleukin-18 (IL-18), and interleukin-6 (IL-6) after MI.^38^ Anti-inflammatory cytokines, such as interleukin-4, interleukin-10, and transforming growth factor beta (TGF-β), are relatively few, resulting in a network imbalance between proinflammatory and anti-inflammatory factors.^39^ Excessive activation of the immune system and persistent inflammatory response increased myocardial damage, promoted ventricular remodeling, and accelerated deterioration of cardiac function after MI.^40,41^ Recent research has shown that immunomodulation was at the top place among various paracrine mechanisms of MSCs therapeutic effect in MI.^6,42^ MSCs were shown to inhibit production of proinflammatory cytokines TNF-α, IL-6, and IFN-γ in some studies.^43,44^ Meanwhile, MSCs were found to stimulate production of anti-inflammatory cytokines IL-10 and IL-12.^9,45^ By doing so, MSCs restrict the local inflammation and minimize the cardiac tissue damage.^46^ In the treatment of MI with BMSCs combined with CS hydrogel, our results have been shown to inhibit the secretion of proinflammatory factors such as IL-6, TNF-α, IL-1β, IL-18, caspase-11, and caspase-1. Based on BMSCs-mediated immunosuppressive effects, vascular endothelial cells were protected, ventricular remodeling was ameliorated, and myocardial function recovery was promoted.

Numerous inflammatory signals in the inured environment will trigger a programmed cell death for pyroptosis.^47^ Pyroptosis is believed to be mediated by 2 cysteine-containing aspartate proteolytic enzymes (caspase), including caspase-1 and caspase-11.^13,48^ The caspase family includes more than a dozen members, most of which function in apoptosis; however, caspase-1 and caspase-11 are inflammatory caspase.^49,50^ In an inflammatory environment, the NLRP3 inflammasome is activated, which in turn regulates the activation of caspase-1.^51,52^ Caspase-1 promotes the maturation of the cytokines pro-IL-1β and pro-IL-18 during the process of innate immune defense.^47,48^ At the same time, caspases-1 activates the GSDMD protein by cleavage of the aminoterminal and carboxyterminal linkers of GSDMD.^28^ The GSDMD binds to lipids, forming pores in the cell membrane, and eventually causes the cells to gradually expand until it ruptures and causes death.^53^ With the release of mature IL-1β and IL-18, a strong inflammatory response is activated.^54^ In this study, we found that transplanted BMSCs could reduce the pyroptosis of vascular endothelial cells by inhibiting the inflammatory response, and we speculated that this effect was achieved by the paracrine effect of BMSCs. Subsequently, we utilized CM of BMSCs to culture the pyroptosis of HUVECs induced by LPS and ATP in vitro. It was found that the CM of BMSCs could effectively inhibit the expression of pyroptosis-related genes and proteins (IL-β, IL-18, caspase-1, NLRP3, GSDMD, and ICAM-1).

## CONCLUSIONS

Local application of transplanted BMSCs with CS hydrogel significantly improved the heart function, ameliorated the ventricular remodeling, and promoted the neovasculature formation by regulating inflammatory microenvironment. This approach provides a novel revenue for effective application of BMSCs transplantation in MI treatment. Although the underneath mechanisms require further clarification, the impact of BMSCs alleviated the pyroptosis of vascular endothelial cells by inhibiting the inflammatory responses via the paracrine mechanism seems to be especially relevant.

## Supplementary Material

SUPPLEMENTARY MATERIAL
