# Supplementary material for: Chitosan Hydrogel Enhances the Therapeutic Efficacy of Bone Marrow–Derived Mesenchymal Stem Cells for Myocardial Infarction by Alleviating Vascular Endothelial Cell Pyroptosis
Source: J Cardiovasc Pharmacol. 2019 Nov 11;75(1):75–83. doi: 10.1097/FJC.0000000000000760 (PMC7668671; doi:10.1097/FJC.0000000000000760)
Supplement: SUPPLEMENTARY MATERIAL [file jcvp-75-75-s001.docx]

**Supplementary Information:**

**Chitosan Hydrogel Enhances the Therapeutic Efficacy of BMSCs for Myocardial Infarction by Alleviating Vascular Endothelial Cell Pyroptosis**

Yang Liu, Panyang Li, Chenhui Qiao, Tiejun Wu, Xiaoke Sun, Meng Wen, Weihua Zhang

**Supplemental Table 1**: Primers used for real-time PCR

| \| Gene \| Forward primer \| Reverse primer \| \| --- \| --- \| --- \| \| mEGF \| TTGACAAGTGGCAGGAGGTC \| ATCCCTACGTCCGTCCAGAA \| \| mPGF \| ACTTGGGAACACAAGAAGCCT \| CTCTCCAGAGCCGAATGTCC \| \| mHGF \| TTCTTTCAGCCCGGCATCTC \| CAAGAACTTGTGCCGGTGTG \| \| mIGF-1 \| GCATACCTGCCTGGGTGTC \| TTTGTCGATAGGGACGGGGA \| \| mTNF-α \| ACAGAAAGCATGATCCGCG \| GCCCCCCATCTTTTGGG \| \| mIL-6 \| GAGGATACCACTCCCAACAGACC \| AAGTGCATCATCGTTGTTCATACA \| \| mIL-18 \| AGCAGTGGTTTTCAGCTGGG \| GGCAGGAGTCCAGAAAGCAT \| \| mCaspase-11 \| TGCAGAGCTATTACTCGCGG \| GAGGCTTGGACGTGACTTGA \| \| mCaspase-1 \| GAAACGCCATGGCTGACAAG \| ACTTGAGGGTCCCAGTCAGT \| \| mGAPDH \| CCCTTAAGAGGGATGCTGCC \| ACTGTGCCGTTGAATTTGCC \| \| hIL-1β \| CAACCAACAAGTGATATTCTCCATG \| GATCCACACTCTCCAGCTGCA \| \| hIL-18 \| AAGATGGCTGCTGAACCAGT \| GAGGCCGATTTCCTTGGTCA \| \| hCaspase-1 \| ATGGCCGACAAGGTCCTC \| TTAATGTCCTGGGAAGAGGTA \| \| hNLPR3 \| GCTGGCATCTGGGGAAACCT \| CTTAGGCTTCGGTCCACACA \| \| hGSDMD \| CTCGCCGACTTCCGTAAACT \| TCCAGCGATCCTGGGTTCTA \| \| hICAM-1 \| TCTTCCTCGGCCTTCCCATA \| AGGTACCATGGCCCCAAATG \| \| hGAPDH \| AATGGGCAGCCGTTAGGAAA \| GCGCCCAATACGACCAAATC \| |
| --- | --- | --- | --- | --- | --- | --- | --- | --- | --- | --- | --- | --- | --- | --- | --- | --- | --- | --- | --- | --- | --- | --- | --- | --- | --- | --- | --- | --- | --- | --- | --- | --- | --- | --- | --- | --- | --- | --- | --- | --- | --- | --- | --- | --- | --- | --- | --- | --- | --- | --- | --- | --- | --- | --- |

**Supplemental Figure:**


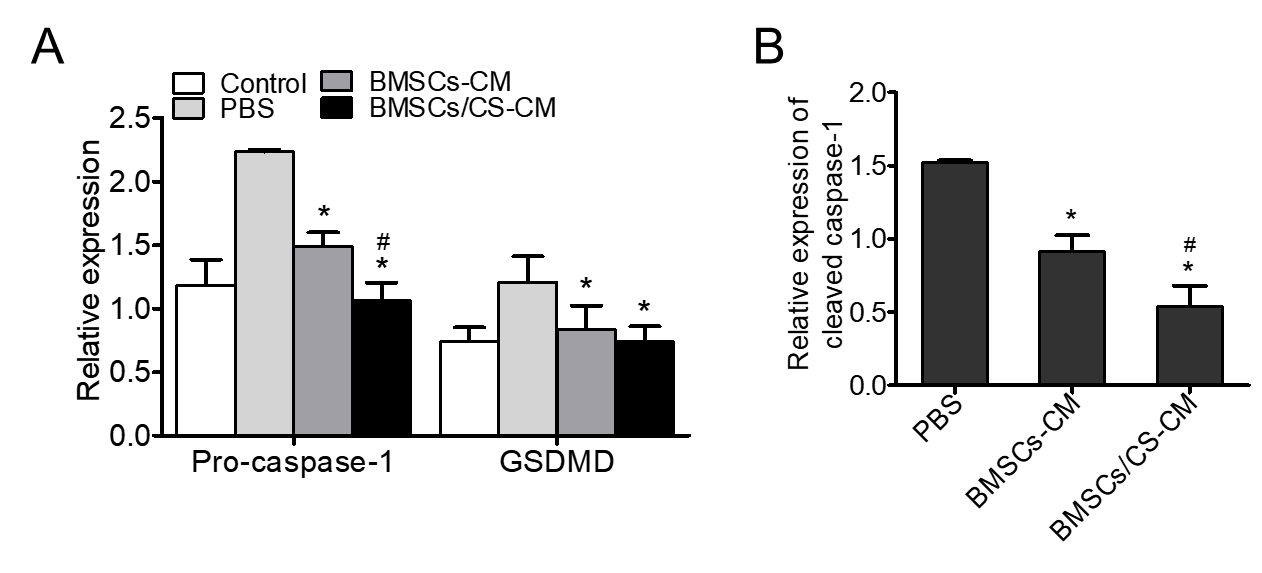


**Supplemental Figure 1.** (A) Quantitative data of the expression of Pro-caspase-1 and GSDMD. (B) Quantitative data of the expression of Cleaved-caspase-1. Data are expressed as mean ± SEM. ^*^P < 0.05 versus PBS; ^#^P < 0.05 versus BMSCs-CM. All experiments were performed in triplicate. BMSCs-CM, BMSCs conditioned medium; BMSCs/CS-CM, conditioned medium of co-culture BMSCs with CS hydrogel.
